# Critical appraisal of “Fruquintinib in less pretreated patients: multivariate profile-matching analysis of FRESCO-2 to FRESCO”

**DOI:** 10.1097/MS9.0000000000004438

**Published:** 2025-12-03

**Authors:** Asra Amjad, Muddassir Khalid

**Affiliations:** a Islamic International Medical College, Riphah International University, Rawalpindi, Pakistan; b Department of Medicine, Nishtar Medical University, Multan, Pakistan

*Dear Editor,*

I appreciate Dasari *et al* for their enlightening multivariate profile-matching research that contrasts FRESCO-2 with FRESCO and provides positive information on the effectiveness of fruquintinib in patients with metastatic colorectal cancer (mCRC) who have received less treatment^[1]^. It is timely and admirable to try to reduce regional and temporal differences among trial populations. However, a number of limitations still need to be addressed before these results can significantly alter clinical paradigms.

First, the development of personalized therapy is hampered by the lack of biomarker-stratified subgroup analyses, especially with regard to VEGF/VEGFR polymorphisms or tumor microenvironment signals. Given that anti-angiogenic efficacy varies among molecular subtypes, biomarker-driven selection may be a more effective way to fine-tune the benefits of treatment^[2]^.

Second, although acknowledged, the confounding effects of previous therapeutic exposures were not adequately addressed. Given the sequencing sensitivity of anti-angiogenic agents, the observed effect size of fruquintinib in earlier lines may be distorted by differences in the two trial populations’ prior use of regorafenib and TAS-102^[3]^.

Third, the significantly higher treatment discontinuation rate in the matched FRESCO-2 subset (22.5% vs 15.1%) raises concerns about regional variations in supportive care practices, even though the overall safety profiles seem to be consistent^[4]^. These practical variations are worth further investigation since they are becoming more significant in the treatment of refractory mCRC.

Lastly, even though quality-of-life (QoL) data were gathered in FRESCO-2, it is a lost opportunity that they were not included in this analysis. QoL measures are crucial for assessing therapeutic benefit in a treatment-refractory context because improvements in progression-free or overall survival are frequently small^[5]^.

The integration of validated biomarkers, harmonized real-world evaluations across health systems, and investigation of fruquintinib’s placement within more comprehensive treatment sequences should, in my opinion, be the top priorities of future research.

This manuscript is in compliant to TITAN Guidelines, 2025, declaring no use of AI^[6]^.

## Data Availability

Not applicable.
